# RETRACTION: Risk Factors Associated With Surgical Site Infections in Patients Undergoing Cardiothoracic Surgery: A Systematic Review and Meta‐Analysis

**DOI:** 10.1111/iwj.70526

**Published:** 2025-04-18

## Abstract

**RETRACTION**: ZhangY. , TanS. , ChenS. , and FanX. , “Risk Factors Associated With Surgical Site Infections in Patients Undergoing Cardiothoracic Surgery: A Systematic Review and Meta‐Analysis,” International Wound Journal 21, no. 4 (2024): e14573, 10.1111/iwj.14573.38102858 PMC10961885

The above article, published online on 15 December 2023, in Wiley Online Library (http://onlinelibrary.wiley.com/), has been retracted by agreement between the journal Editor in Chief, Professor Keith Harding; and John Wiley & Sons Ltd. Following an investigation by the publisher, all parties have concluded that this article was accepted solely on the basis of a compromised peer review process. The editors have therefore decided to retract the article. The authors did not respond to our notice regarding the retraction.

**RETRACTION**: Y. Zhang , S. Tan , S. Chen , and X. Fan , “Risk Factors Associated With Surgical Site Infections in Patients Undergoing Cardiothoracic Surgery: A Systematic Review and Meta‐Analysis,” International Wound Journal 21, no. 4 (2024): e14573, 10.1111/iwj.14573.38102858 PMC10961885

The above article, published online on 15 December 2023, in Wiley Online Library (http://onlinelibrary.wiley.com/), has been retracted by agreement between the journal Editor in Chief, Professor Keith Harding; and John Wiley & Sons Ltd. Following an investigation by the publisher, all parties have concluded that this article was accepted solely on the basis of a compromised peer review process. The editors have therefore decided to retract the article. The authors did not respond to our notice regarding the retraction.

